# Photosensitivity to Triflusal: Formation of a Photoadduct with Ubiquitin Demonstrated by Photophysical and Proteomic Techniques

**DOI:** 10.3389/fphar.2016.00277

**Published:** 2016-08-29

**Authors:** Edurne Nuin, Dolores Pérez-Sala, Virginie Lhiaubet-Vallet, Inmaculada Andreu, Miguel A. Miranda

**Affiliations:** ^1^Instituto de Tecnología Química, Universitat Politècnica de València-Consejo Superior de Investigaciones CientíficasValencia, Spain; ^2^Departamento de Biología Físico-Química, Centro de Investigaciones Biológicas, Consejo Superior de Investigaciones CientíficasMadrid, Spain; ^3^Unidad Mixta de Investigación IIS La Fe-UPV, Hospital Universitari i Politècnic La FeValencia, Spain

**Keywords:** covalent binding to protein, fluorescence, laser flash photolysis, lysine, mass spectrometry, metabolite, photoallergy

## Abstract

Triflusal is a platelet aggregation inhibitor chemically related to acetylsalicylic acid, which is used for the prevention and/or treatment of vascular thromboembolisms, which acts as a prodrug. Actually, after oral administration it is absorbed primarily in the small intestine, binds to plasma proteins (99%) and is rapidly biotransformed in the liver into its deacetylated active metabolite 2-hydroxy-4-trifluoromethylbenzoic acid (HTB). In healthy humans, the half-life of triflusal is *ca.* 0.5 h, whereas for HTB it is *ca*. 35 h. From a pharmacological point of view, it is interesting to note that HTB is itself highly active as a platelet anti-aggregant agent. Indeed, studies on the clinical profile of both drug and metabolite have shown no significant differences between them. It has been evidenced that HTB displays ability to induce photoallergy in humans. This phenomenon involves a cell-mediated immune response, which is initiated by covalent binding of a light-activated photosensitizer (or a species derived therefrom) to a protein. In this context, small proteins like ubiquitin could be appropriate models for investigating covalent binding by means of MS/MS and peptide fingerprint analysis. In previous work, it was shown that HTB forms covalent photoadducts with isolated lysine. Interestingly, ubiquitin contains seven lysine residues that could be modified by a similar reaction. With this background, the aim of the present work is to explore adduct formation between the triflusal metabolite and ubiquitin as model protein upon sunlight irradiation, combining proteomic and photophysical (fluorescence and laser flash photolysis) techniques. Photophysical and proteomic analysis demonstrates monoadduct formation as the major outcome of the reaction. Interestingly, addition can take place at any of the ε-amino groups of the lysine residues of the protein and involves replacement of the trifluoromethyl moiety with a new amide function. This process can in principle occur with other trifluoroaromatic compounds and may be responsible for the appearance of undesired photoallergic side effects.

## Introduction

**FIGURE 1:**
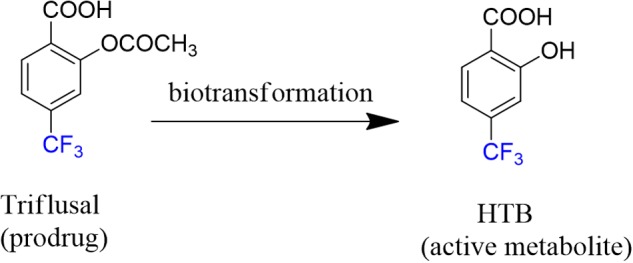
**Chemical structures of triflusal and its metabolite HTB**.

Triflusal (**Figure 1**) is a platelet aggregation inhibitor chemically related to acetylsalicylic acid, which is used for the prevention and/or treatment of vascular thromboembolisms (McNeely and Goa, 1998; Gonzalez-Correa and De La Cruz, 2006). Additionally, triflusal increases nitric oxide synthesis in neutrophils, leading to an increased vasodilator potential (Matías-Guiu et al., 2003).

From a clinical standpoint, triflusal shows similar efficacy to aspirin in preventing stroke, but the former is associated with a reduced risk of hemorrhagic complications. Structurally, it differs from the latter in having a trifluoromethyl moiety at position 4 (Matías-Guiu et al., 2003; Gonzalez-Correa and De La Cruz, 2006).

Actually, triflusal acts as a prodrug since after oral administration, it is absorbed primarily in the small intestine, binds to plasma proteins (99%) and is rapidly biotransformed in the liver into its deacetylated active metabolite 2-hydroxy-4-trifluoromethylbenzoic acid (HTB, **Figure 1**) (Ramis et al., 1991; McNeely and Goa, 1998; Cho et al., 2003) In healthy humans, the half-life of triflusal is *ca.* 0.5 h, whereas for HTB it is *ca*. 35 h (Gonzalez-Correa and De La Cruz, 2006). From a pharmacological point of view, it is interesting to note that HTB is itself highly active as a platelet anti-aggregant agent. Indeed, studies on the clinical profile of both drug and metabolite have shown no significant differences between them (Ramis et al., 1991).

Although their side effects are mainly gastrointestinal, it has been evidenced that both triflusal and HTB display ability to induce photoallergy in humans (Serrano et al., 1987; Lee et al., 1999, 2001; Nagore et al., 2000). This phenomenon involves a cell-mediated immune response, which is initiated by covalent binding of a light-activated photosensitizer (or a species derived therefrom) to a protein, a process known as haptenation (Ariza et al., 2011). Interestingly, although aspirin presents a similar chemical structure, it is not associated with photosensitivity disorders. Therefore, as mentioned above, the trifluoromethyl group at position 4 present in triflusal and HTB should be responsible for the photosensitizing properties of the molecule (Bosca et al., 2001; Caffieri et al., 2007; Montanaro et al., 2009).

Ubiquitin is a small (8.5 KDa) regulatory protein, with 76 amino acids, present in all eukaryotic cells, which was discovered in the early 1970s (Pickart and Eddins, 2004; Herrmann et al., 2007; Hochstrasser, 2009).

Covalent ubiquitination is a major regulatory post-translational process, involving attachment of the ubiquitin to lysine residue/s on a substrate protein or on another ubiquitin molecule, leading to mono or polyubiquitination (Dikic et al., 2009; Suryadinata et al., 2014). Thus, monobiquitination can modify protein activity and localization by endocytosis, cell-cycle control or lysosomal targeting (Dikic et al., 2009), whereas polyubiquitination is implicated in DNA repair and immune signaling (Dikic et al., 2009; McIntyre and Woodgate, 2015). Besides, the discovery that ubiquitin chains target proteins to the proteasome, which degrades and recycles proteins, was honored with the Nobel Prize in chemistry in 2004.

Moreover, ubiquitin does not have a well-defined active site although it binds to the so-called ubiquitin binding sites, which are modular protein domains that non-covalently bind ubiquitin (Hicke et al., 2005; Hurley et al., 2006; Dikic et al., 2009) and reveal information about the functionality and the mechanism of intermolecular regulation.

In this context, small proteins like ubiquitin could be appropriate models for investigating covalent binding by means of MS/MS and peptidic fingerprint analysis (Jeram et al., 2009; Hong et al., 2015; Ramirez et al., 2015). In previous work, it was shown that HTB forms covalent photoadducts with isolated lysine and polylysine (Montanaro et al., 2009). Interestingly, ubiquitin contains seven lysine residues (Lys6, Lys11, Lys27, Lys29, Lys33, Lys48, and Lys 63) that could be modified by a similar reaction.

With this background, the goal of the present work is to identify possible adduct formed between the triflusal metabolite and ubiquitin as model protein, upon sunlight irradiation, combining proteomic and photophysical (fluorescence and laser flash photolysis) techniques.

## Materials and Methods

### Materials and Solvents

HTB was purchased from WAKO (Osaka, Japan) and was used without further purification; *n*-butylamine and bovine ubiquitin, whose sequence is identical to the human protein, were provided from Sigma–Aldrich (Steinheim, Germany). Phosphate buffered saline solution (PBS, pH = 7.4, 0.01 M) was prepared by dissolving Sigma tablets in the appropriate amount of deionized water. Sephadex G-25 columns (PD-10) were acquired from Amersham Pharmacia Biotech (UK). Dichloromethane and ethyl acetate were from Scharlab (Sentmenat, Spain).

### Photoaddition of HTB to Ubiquitin

Solutions containing HTB (5 × 10^-5^ M) and ubiquitin (5 × 10^-5^ M) in PBS were incubated 1 h in the dark. Samples were then irradiated for 1 h (18.9 J/cm^2^) under the sunlight. For all photophysical studies, the photoadduct was separated from the protein using guanidine chloride and disposable Sephadex G-25 columns (PD-10) (Amersham Pharmacia Biotech, UK) equilibrated with PBS and the final product was further lyophilized at -55°C for 16 h. Control included drug–protein mixture kept in the dark and ubiquitin without irradiation.

### Synthesis of HTB-*n*-Butylamine Adduct

A mixture of HTB (150 mg, 0.73 mmol) and *n*-butylamine (720 μL, 7.3 mmol) dissolved in deaerated PBS solution (20 mL) was placed in quartz tubes and irradiated overnight by means of a multilamp photoreactor equipped with six lamps (Hitachi, F15T8/BL) with a maximal output at *ca*. 300 nm (Gaussian distribution). The crude product was dissolved in ethyl acetate, washed with 1 M HCl and brine, dried over MgSO_4_ and concentrated under vacuum. The residue was washed with dichloromethane several times to get HTB-butylNH_2_ as a white solid (94 mg, 63%). ^1^H NMR (300 MHz, CD_3_OD): *δ* 0.98 (t, *J* = 7.3 Hz, 3H), 1.38–1.45 (m, 2H), 1.56–1.66 (m, 2H), 3.32–3.40 (m, 2H), 7.29 (dd, *J* = 8.2 Hz and 1.6 Hz, 1H), 7.34 (d, *J* = 1.6 Hz, 1H), 7.93 (d, *J* = 8.2 Hz, 1H); ^13^C NMR (75 MHz, CD_3_OD): *δ* 14.2, 21.2, 32.5, 40.9, 116.4, 117.0, 118.5, 131.9, 142.4, 163.0, 169.1, 173.0. Exact mass: m/z found, 236.0922 calculated for C_12_H_14_NO_4_ (M-H)^+^ 236.0928.

### Laser Flash Photolysis Experiments

Laser flash photolysis (LFP) experiments were carried out with a pulsed XeCl excimer laser (α_exc_ = 308 nm, *ca*. 17 ns pulse width, <100 mJ per pulse). In general, samples received between 1 and 3 pulses for all the kinetic experiments. A pulsed Lo255 Oriel Xenon lamp was used as detecting light source. The observation wavelength was selected with a 77200 Oriel monochromator, and the signal amplified by an Oriel photomultiplier tube (PMT) system made up of a 77348 side-on tube, 70680 housing and a 70705 power supply. The signal was registered with a TDS-640A Tektronix oscilloscope and subsequently transferred to a personal computer. The absorbance of the solutions was adjusted at *ca.* 0.21 at the excitation wavelength. All transient spectra were recorded at room temperature using 10 mm × 10 mm quartz cells with 4 mL capacity and were bubbled for 15 min with N_2_ before acquisition.

### Fluorescence Measurements

Steady-state fluorescence experiments were carried out using a Photon Technology International (PTI, Germany) LPS-220B spectrofluorometer, equipped with a monochromator in the range of 200–700 nm. Time-resolved fluorescence measurements were performed with a Time Master fluorescence lifetime spectrometer TM 2/2003 from PTI, using a hydrogen/nitrogen flash lamp as the excitation source. The kinetic traces were fitted by monoexponential decay functions, using a deconvolution procedure to separate them from the lamp pulse profile. Emission measurements were performed in the region of 330–600 nm. The absorbance of the solutions was adjusted at ca. 0.08 at 308 nm. All measurements were performed at room temperature using 10 mm × 10 mm quartz cells of 4 mL capacity, under aerobic conditions.

### Mass Spectrometry Analysis of HTB-Modified Ubiquitin by MALDI-TOF

Solutions containing HTB (5 × 10^-5^ M) and ubiquitin (5 × 10^-6^ M) in PBS were incubated 1 h in the dark, after which they were exposed to sunlight (doses of UVA light of 18.9 J/cm^2^). Incubation mixtures were directly mixed with the matrix and applied to the MALDI-TOF analysis on an Autoflex III MALDI-TOF-TOF mass spectrometer (Bruker), operated in the positive mode as previously described (Renedo et al., 2007; Oeste et al., 2011).

Steady-state fluorescence (α_exc_ = 308 nm) of phosphate buffer solution of HTB (black line), sunlight irradiated HTB-ubiquitin (1:1) after sephadex filtration (green line) or non-irradiated HTB-ubiquitin (1:1) after sephadex filtration (blue line), and HTB-butylNH_2_ adduct (red line). **(Bottom)** Corresponding decays.

### Protein Digestion and LC-ESI-MS/MS Analysis

Solution containing HTB (5 × 10^-5^ M) and ubiquitin (5 × 10^-6^ M) in PBS was incubated 1 h in the dark and irradiated by sunlight. The samples were enzymatically digested into smaller peptides using trypsin. Subsequently, these peptides were analyzed using nanoscale liquid chromatography coupled to tandem mass spectrometry (nano LC-MS/MS). Briefly, 20 μg of sample were taken (according to Qubit quantitation) and the volume was set to 20 μL. Digestion was achieved with sequencing grade trypsin (Promega, trypsin: protein ratio 1:20 w/w) V = 64 μL, overnight 37°C. Digestion was stopped with 7 μL 10% TFA (trifluoroacetic acid). The final peptide mixture was at a concentration *ca* 0.5 μg/μL.

Next, 5 μL of sample were loaded onto a trap column (NanoLC Column, 3 μ C18 CL, 100 umx 15 cm; Nikkyo) and desalted with 0.1% TFA at 2 μL/min during 10 min. The peptides were then loaded onto an analytical column (LC Column, 3 μ C18 CL, 75 umx12 cm, Nikkyo) equilibrated in 5% acetonitrile 0.1% formic acid. Elution was carried out with a linear gradient of 5–40% B in A for 30 min (A: 0.1% formic acid; B: acetonitrile, 0.1% formic acid) at a flow rate of 300 nL/min. Peptides were analyzed in a mass spectrometer nanoESI qQTOF (5600 TripleTOF, ABSCIEX). The tripleTOF was operated in information-dependent acquisition mode, in which a 0.25-s TOF MS scan from 350 to 1250 m/z was performed, followed by 0.05-s product ion scans from 100 to 1500 m/z on the 10 most intense 2–5 charged ions.

ProteinPilot v4.5. (ABSciex) search engine default parameters were used to generate peak list directly from 5600 TripleTOF wiff files. The obtained mgf was used for identification with MASCOT (v 4.0, Matrix- Science). Database search was performed on Home Made (includes sequence of interest and the contaminants described in Expasy). Searches were done with tryptic specificity allowing one missed cleavage and a tolerance on the mass measurement of 75 ppm in MS mode and 0.6 Da in MS/MS mode. Oxidation of Met and deamidation of Asn and Gln as variable modifications. HTB modification was set as variable for K, S, T.

## Results and Discussion

Sunlight irradiation of buffered aqueous solutions of HTB (5 × 10^-5^ M, **Figure 1**) and ubiquitin (1:1 molar ratio) was performed at noon in Valencia (Spain, July). Then, the protein material was separated from the free metabolite by gel-filtration chromatography (Sephadex). The high-molecular weight fraction was examined spectroscopically to reveal the presence of a covalently linked chromophore. As a control a 1:1 solution of HTB:ubiquitin was kept in the dark and filtered by Sephadex. This way the comparison between irradiated and non-irradiated samples would inform about the formation of a covalent adduct between the metabolite and the protein.

**FIGURE 2:**
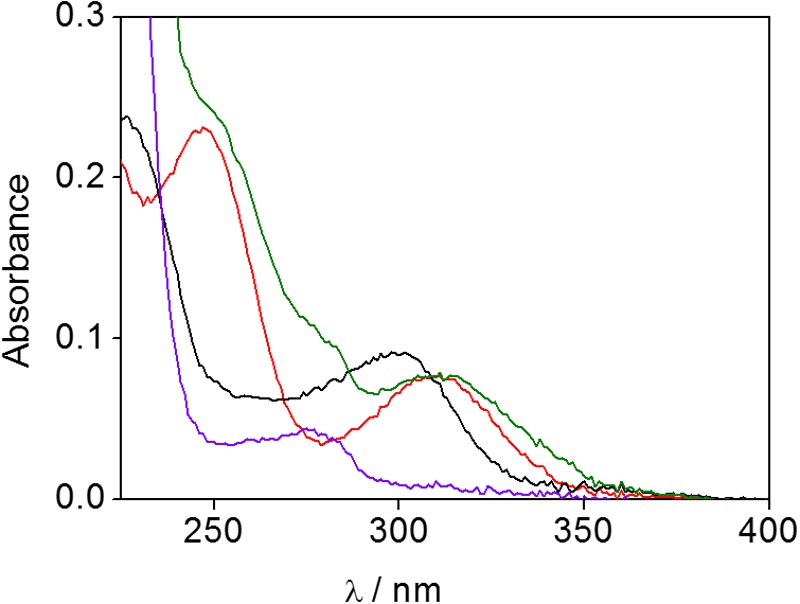
**UV-Vis spectra of phosphate buffer solutions of HTB (black), ubiquitin (violet), HTB-ubiquitin adduct (green) and HTB-ButylNH_2_ adduct (red)**.

A first approach was based on UV-Vis spectrophotometry, so the absorption spectrum of the obtained proteinaceous fraction was registered together with those of ubiquitin and HTB for comparison. The protein alone in solution showed a band with a maximum at 270 nm and no significant absorption at α>300 nm (**Figure 2**, violet line); by contrast HTB displayed a UVB-band centered at 300 nm (**Figure 2**, black line). Interestingly, the irradiated and filtered HTB:ubiquitin solution clearly exhibited an absorption peaking at 310 nm (**Figure 2**, green line), whereas no UVB-band was observed for the non-irradiated and filtered sample (see Supplementary Material). This result points toward the formation of a covalent adduct between the protein and the metabolite.

**FIGURE 3:**
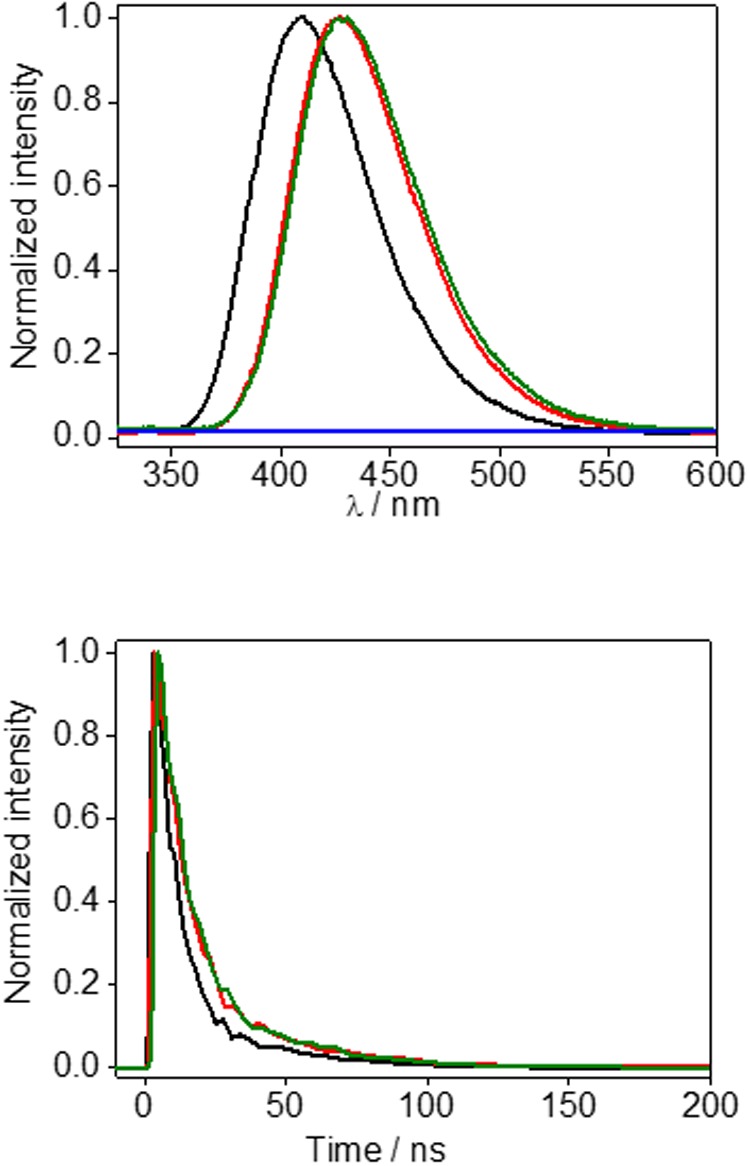
**(Top)** Steady-state fluorescence (α_exc_ = 308 nm) of phosphate buffer solution of HTB (black line), sunlight irradiated HTB-ubiquitin (1:1) after sephadex filtration (green line) or non-irradiated HTB-ubiquitin (1:1) after sephadex filtration (blue line), and HTB-butylNH_2_ adduct (red line). **(Bottom)** Corresponding decays.

To get further insight, steady-state and time-resolved fluorescence experiments were conducted. At the excitation wavelength of 308 nm, HTB emission was detected at *ca*. 405 nm in agreement with previous reports, (Bosca et al., 2001; Montanaro et al., 2009) whereas a red shifted fluorescence spectrum, with α_em_ at *ca.* 420 nm, was obtained for the irradiated and filtered HTB-ubiquitin sample (**Figure 3** top, green line). No emission was detected for the control solution kept in the dark (**Figure 3** top, blue line). Moreover, time-resolved experiments revealed different lifetimes for the HTB and HTB-ubiquitin samples, τ being of *ca*. 9.5 and 13.5 ns, respectively (**Figure 3** bottom, black and green lines).

**FIGURE 4:**
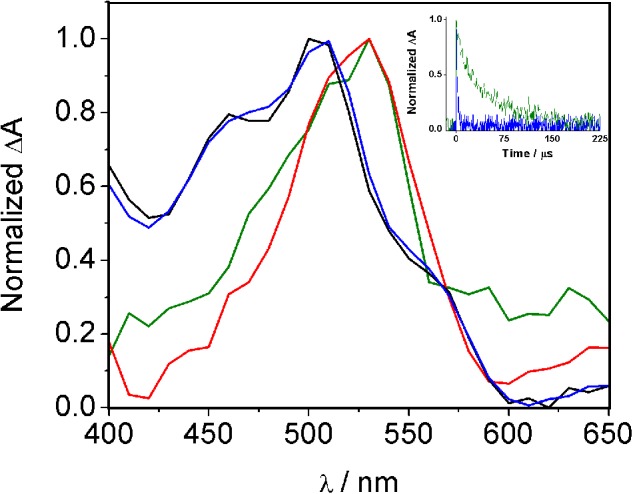
**Normalized transient absorption spectra of HTB (black line), HTB/ubiquitin 1:1 (blue line), HTB-ubiquitin (green line) and HTB-butylNH_2_ (red line) adducts in phosphate buffer N_2_-flushed solutions, 0.5 μs after the laser pulse.** Inset: normalized decay traces of HTB/ubiquitin 1:1 (blue) and HTB-ubiquitin adduct (green).

Additional spectroscopic studies of the covalent adduct between HTB and ubiquitin were performed by nanosecond LFP using a 308 nm XeCl excimer laser for excitation. The transient spectra registered for a nitrogen-flushed solution of HTB alone or irradiated and filtered HTB-ubiquitin in phosphate buffer solutions are shown in **Figure 4**. The HTB-ubiquitin adduct exhibited a transient absorption centered at 520 nm, a wavelength close to that observed for the HTB triplet excited state at α_TT_ of *ca*. 500 nm (Bosca et al., 2001; Montanaro et al., 2009).

Thus, detection of a spectroscopic response for the high molecular weight fraction of the irradiated HTB:ubiquitin solution evidenced the formation of a covalent photoadduct. Furthermore, the spectral similarities between the free metabolite and this proteinaceous fraction indicated the presence of a HTB-like chromophore in the modified ubiquitin biomolecule.

**FIGURE 5:**
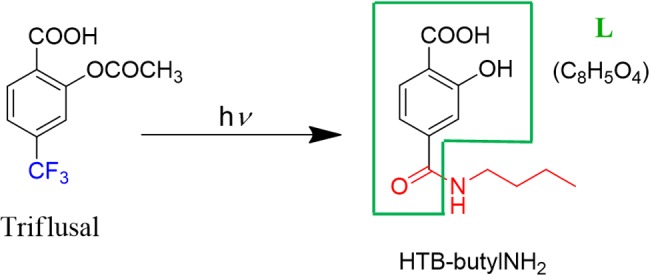
**Photoadduct HTB-butylNH_2_ formation**.

Thus, based on the previous studies that have established photoaddition of HTB at the ε-amino group of lysine, (Montanaro et al., 2009) the model compound (HTB-butylNH_2_, **Figure 5**) was synthesized by UVB-irradiation of a phosphate buffer solution of *n*-butylamine and HTB. Photoadduct HTB-butylNH_2_ was obtained as main product in 63% yield and fully characterized by NMR and HRMS (see Supplementary Material). The spectroscopic characterization of this model compound in phosphate buffer revealed a strong analogy between its photobehavior and that of HTB-ubiquitin photoadduct. Indeed, it displayed the same UVB absorption maximum (**Figure 2**, red line), a coincident fluorescence emission and lifetime (**Figure 3**), as well as an identical transient absorption spectrum (**Figure 4**).

**SCHEME 1:**
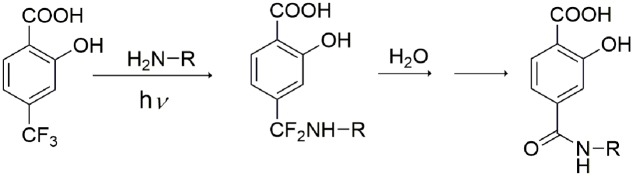
**Proposed mechanism of HTB-ubiquitin adduct formation**.

**FIGURE 6:**
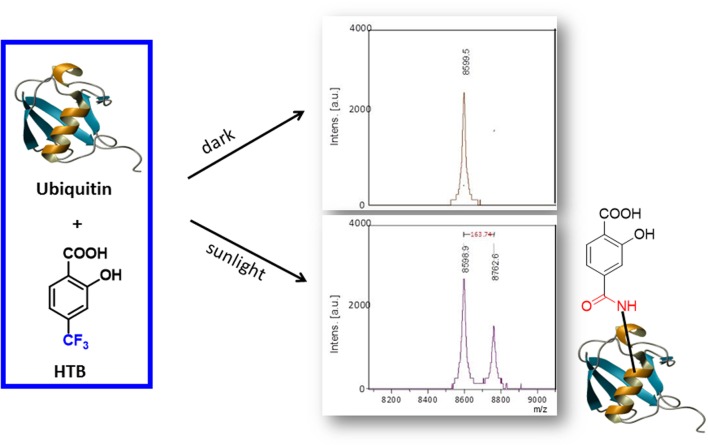
**MALDI-TOF spectra of HTB-ubiquitin (10:1) kept in the dark (Top) or solution after 30 min of sunlight irradiation (Bottom)**.

Altogether, these results support formation of a HTB-ubiquitin adduct through photoaddition at the ε-amino group of lysine (**Scheme 1**). To obtain more precise structural information, the photoreactivity of HTB with the whole ubiquitin biomolecule was addressed by MALDI-TOF and HPLC-nanoESI analysis. Comparison between the MALDI-TOF spectra of sunlight irradiated solutions of ubiquitin alone (5 × 10^-5^ M, *m/z* 8599) and HTB-ubiquitin (10:1) mixture revealed the appearance of a new peak at *m/z* 8763 that corresponds to an increment of 164 amu (**Figure 6**), compatible with L(-H). Similar results were obtained for 1:1 ratio. Next, incubation mixtures were filtered to remove excess HTB and trypsin digestion followed by HPLC-MS/MS was performed in order to investigate the modified peptide sequence and to undertake a detailed characterization of the HTB-ubiquitin adduct. Full scan, as well as fragmentation, data files were analyzed by means of the Mascot^®^ database search engine (Matrix Science, Boston, MA, USA) and by entering variable modifications that take into account the main nucleophilic sites able to react with the trifluoromethyl group of HTB, i.e., Lys, Thr and Ser, (Caffieri et al., 2007; Montanaro et al., 2009). Results confirmed identification of six HTB-ubiquitin derived peptide adducts: _1_MQIFVKTLTGK_11_, _7_TLTGKTITLEVEPSDTIENVK_27_, _12_TITLEVEPSDTIENVKAK_29_, _30_IQDKEGIPPDQQR_42_, _43_LIFAGKQLEDGR_54_, _55_TLSDYNIQKESTLHLVLR_72_ (all peptides have one missed cleavage at Lys), their related data are summarized in **Table 1**.

**Table 1:** Identification of HTB-modified peptide by MS/MS spectrometry.

Peptide adduct ID	Observed precursor ion (m/z)	Charge (z)	Mr (exp)	Mr (calcd)	Sequence	Adduct site
1	715.7316	2	1428.7316	1428.7323	_1_MQIFVK#TLTGK_11_	K6
2	818.0825	3	2451.2257	2451.2268	_7_TLTGK#TITLEVEPSDTIENVK_27_	K11
3	717.6962	3	2150.0668	2150.0630	_12_TITLEVEPSDTIENVK#AK_29_	K27
4	844.3987	2	1686.7828	1686.7849	_30_IQDK#EGIPPDQQR_42_	K33
5	755.8790	2	1509.7434	1509.7463	_43_LIFAGK#QLEDGR_54_	K48
6	765.3953	3	2293.1641	2293.1590	_55_TLSDYNIQK#ESTLHLVLR_72_	K63


**FIGURE 7:**
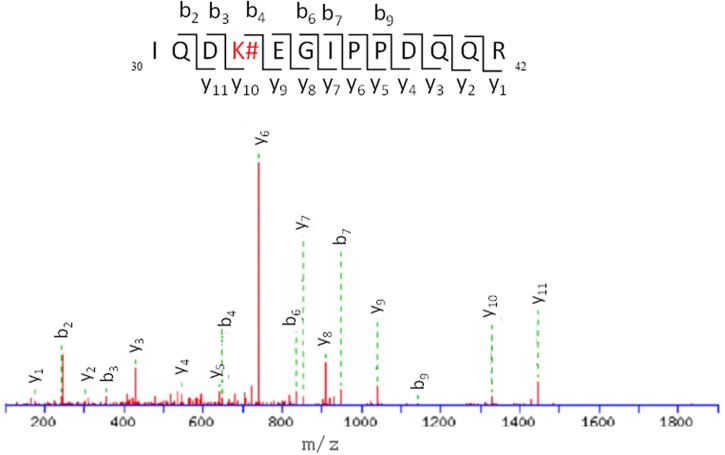
**ESI-MS/MS spectra and fragmentation pathway of the HTB-modified peptide 4**.

Thus, the modification site of each peptide was assessed by tandem mass experiments on the trypsin digests. The MS/MS fragmentation was achieved by selecting the precursor ions given in **Table 1**. The peptide sequence well agreed with the *y* and *b* ion series (see Supplementary Material). Here, the case of fragment 4, namely _30_IQDKEGIPPDQQR_42_, is discussed as an example (**Figure 7**); further information on the others fragments is given in the Supplementary Material. The MS/MS fragment ions showed an unmodified *y* ion series from *y*_1_ to *y*_9_, whereas an increment of *m/z* 292 L(-H)-Lys(-H_2_O) was detected between *y*_9_ to *y*_10_. Accordingly, the *b* ion series suffered the same increase from *b*_3_ to *b*_4_. Thus, the modified amino acid is the Lys 33. Examination of the other five tryptic peptides confirms that Lys is the main site for the adduct formation with modifications detected at K_6_, K_11_, K_27_, K_48_, and K_63_. It should be mentioned that no diagnostic *y* and *b* fragment ions were found for Lys 27 and 29; however, detection of peptide 3 points toward formation of an adduct at site 27, which represents a missed cleavage due to the bulky L substituent that likely hinders trypsin ability to cleave at this site.

## Conclusion

Irradiation of HTB, the active metabolite of triflusal, in the presence of ubiquitin under sunlight gives rise to covalent photobinding. Photophysical and proteomic analysis demonstrate that although the main product found is a monoadduct, the reaction take places at all the ε-amino groups of the lysine residues of the protein and involves replacement of the trifluoromethyl moiety with a new amide function. Concentrations of both drug and protein used in the present work are compatible with those present in blood plasma. Therefore, it is expected that immunologic reactions occur in patients under triflusal therapy in combination with sunlight exposure. This process can in principle occur with other trifluoroaromatic compounds and may be responsible for the appearance of undesired photoallergic side effects.

## Author Contributions

All authors listed, have made substantial, direct and intellectual contribution to the work, and approved it for publication.

## Conflict of Interest Statement

The authors declare that the research was conducted in the absence of any commercial or financial relationships that could be construed as a potential conflict of interest.
