# Supplementary material for: Photosensitivity to Triflusal: Formation of a Photoadduct with Ubiquitin Demonstrated by Photophysical and Proteomic Techniques
Source: Front Pharmacol. 2016 Aug 29;7:277. doi: 10.3389/fphar.2016.00277 (PMC5002410; doi:10.3389/fphar.2016.00277)
Supplement: Supplementary file 1 [file Presentation_1.PDF]

Supplementary Material

Photosensitivity to Triflusal: Formation of a Photoadduct with Ubiquitin Demonstrated by Photophysical and Proteomic Techniques

Edurne Nuin[1], Dolores Pérez-Sala[2], Virginie Lhiaubet-Vallet[1], Inmaculada Andreu[3*], Miguel A. Miranda[1*]

[1]Instituto de Tecnología Química, Universitat Politècnica de València-Consejo Superior de Investigaciones Científicas, Valencia, Spain

[2]Departamento de Biología Físico-Química. Centro de Investigaciones Biológicas, C.S.I.C., Madrid, Spain

[3]Unidad Mixta de Investigación IIS La Fe-UPV, Hospital Universitari i Politècnic La Fe, Valencia, Spain

Index:

S0: Index

S1: UV-Vis spectra of a control solution of HTB and ubiquitin

S2: Fluorescence emission of control solution of HTB and ubiquitin

S3: ^{1}H NMR spectrum of HTB-butylNH_2 (MeOD, 300 MHz)

S4: ^{13}C NMR and DEPT spectra of HTB-butylNH_2 (MeOD, 75 MHz)

S5: ESI-MS/MS spectra and fragmentation pathway of the HTB-modified peptide 1 and 2

S6: ESI-MS/MS spectra and fragmentation pathway of the HTB-modified peptide 3 and 5

S7: ESI-MS/MS spectra and fragmentation pathway of the HTB-modified peptide 6

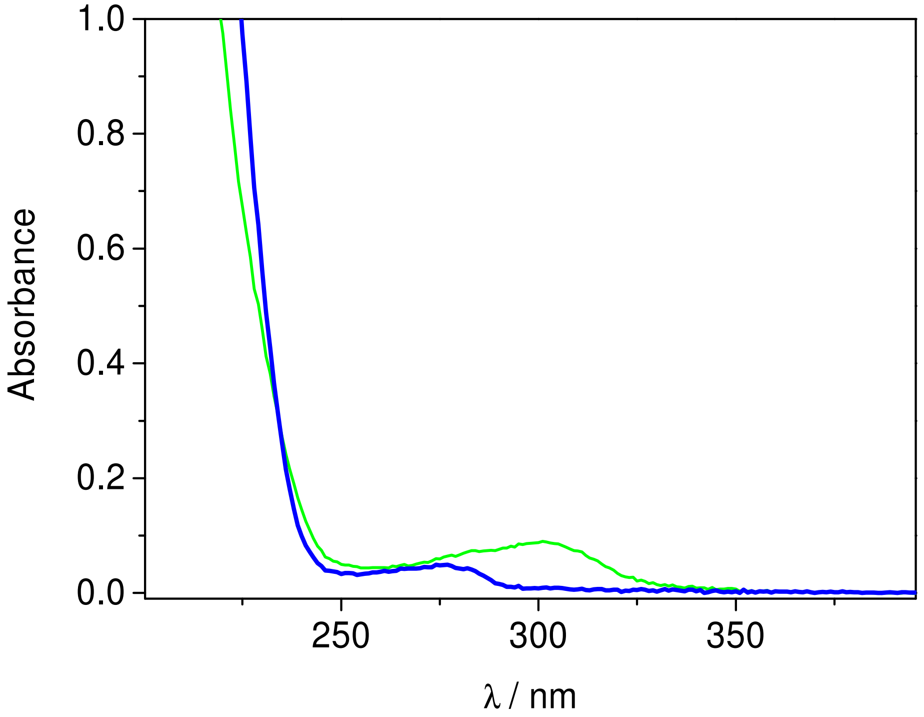

Figure S1. UV-Vis spectra of control phosphate buffer solutions of HTB and ubiquitin (1:1) kept in the dark before (green) and after sephadex filtration (blue)

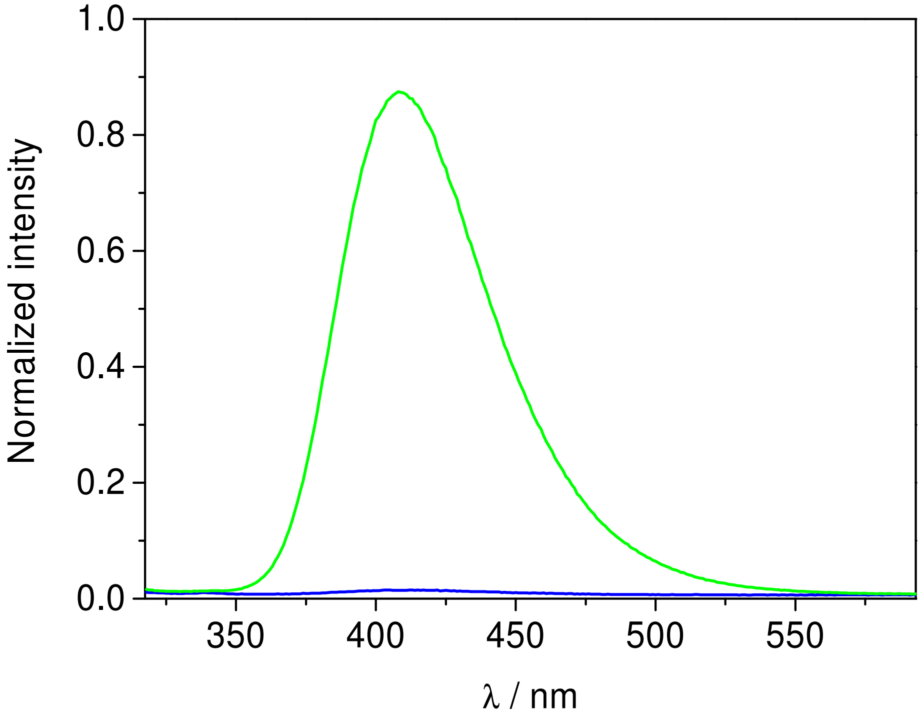

Figure S2. Steady-state fluorescence (λ_{exc} = 308 nm) of control phosphate buffer solution of HTB and ubiquitin (1:1) before (green) and after sephadex filtration (blue)

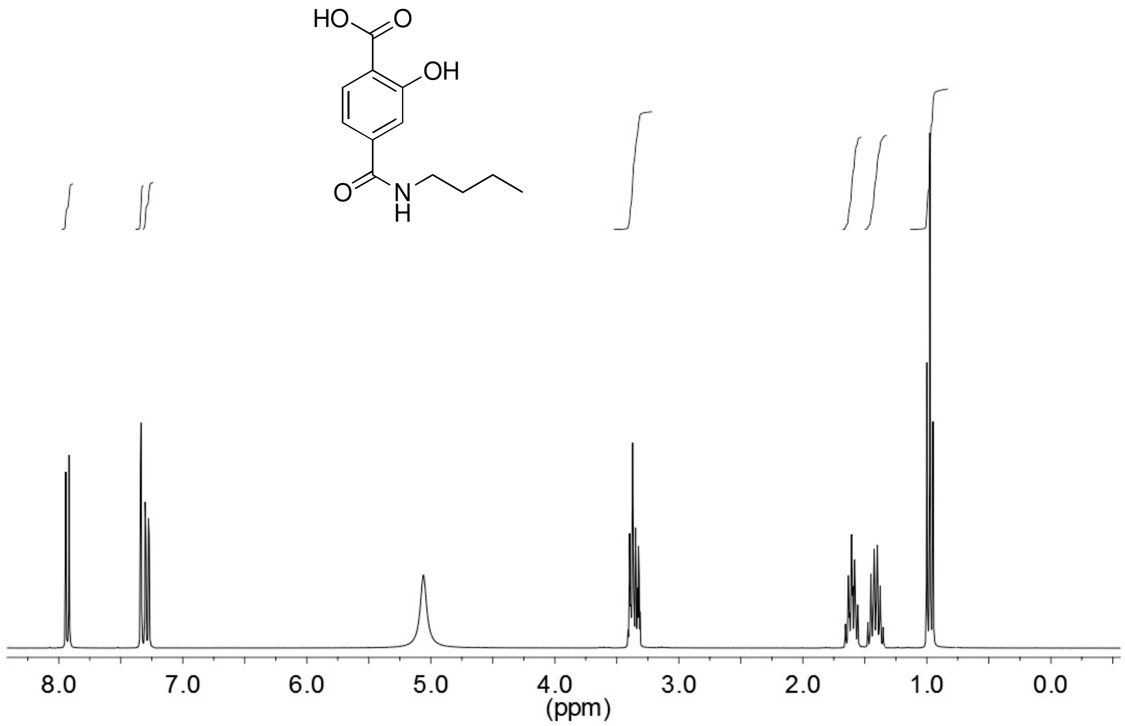

Figure S3. ^{1}H NMR of HTB-butylNH_2 (MeOD, 300 MHz)

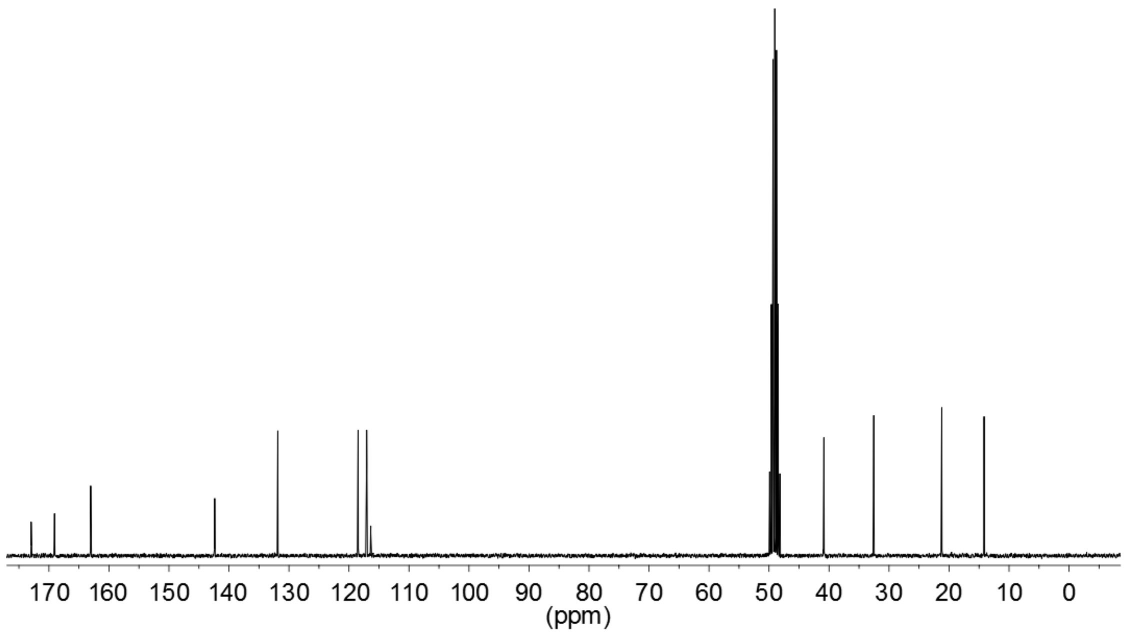

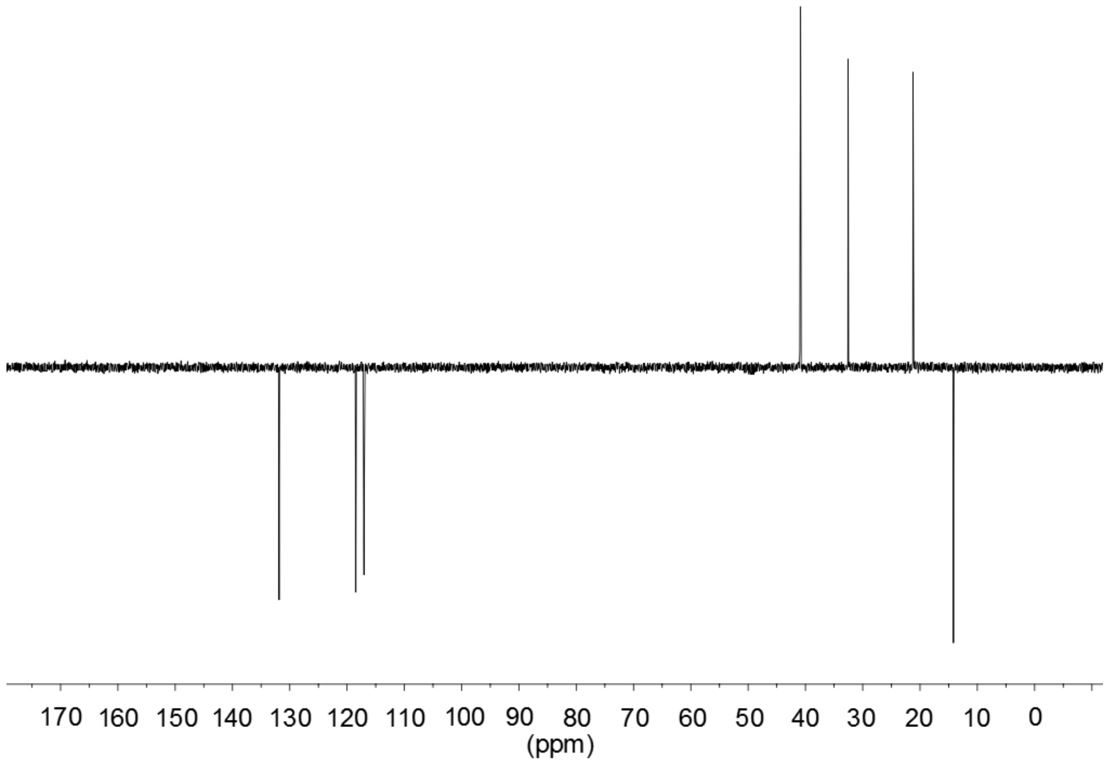

Figure S4. ^{13}C NMR and DEPT spectra of HTB-butylNH_2 (MeOD, 75 MHz)

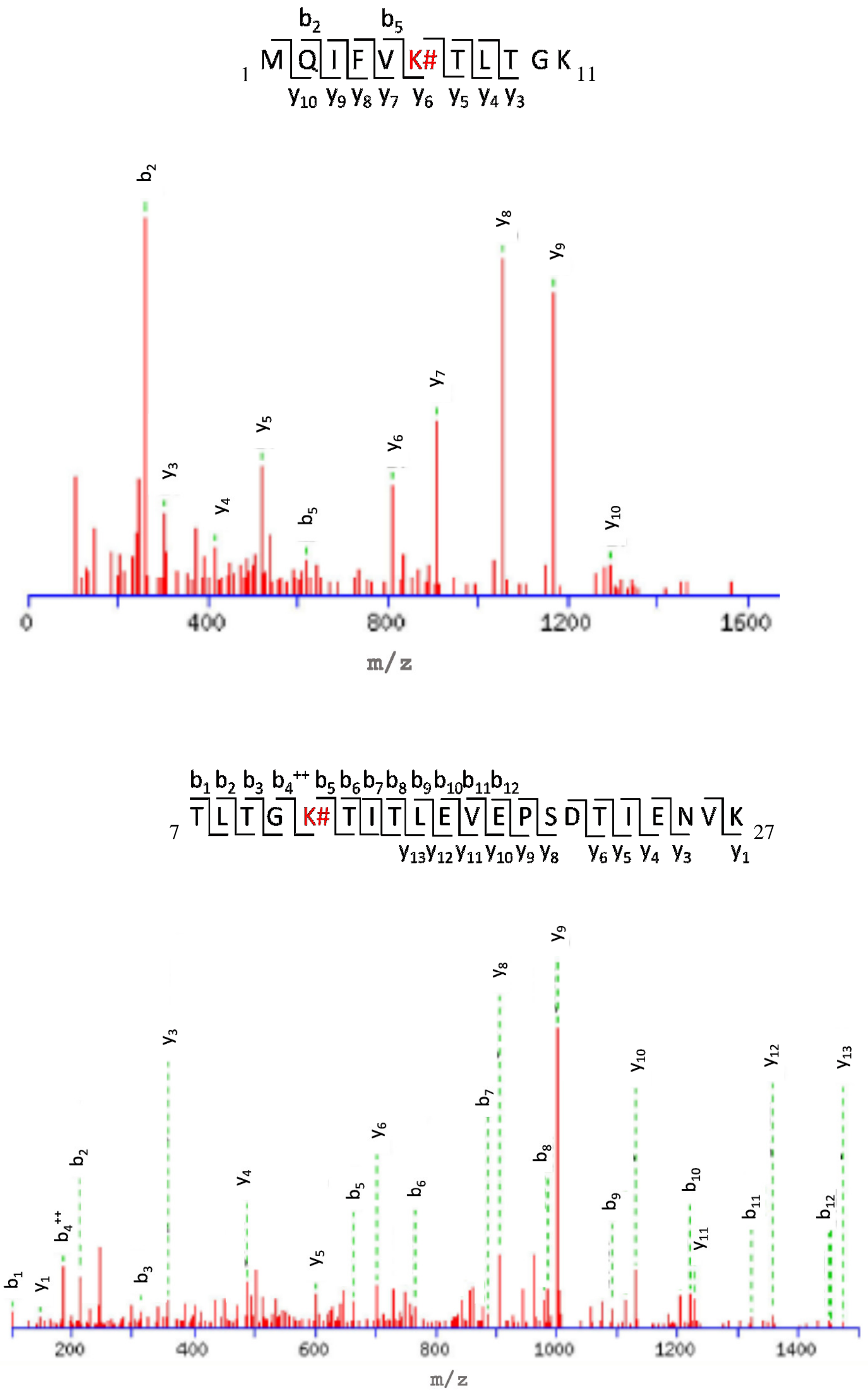

Figure S5. ESI-MS/MS spectra and fragmentation pathway of the HTB-modified peptide 1 (top) and 2 (bottom)

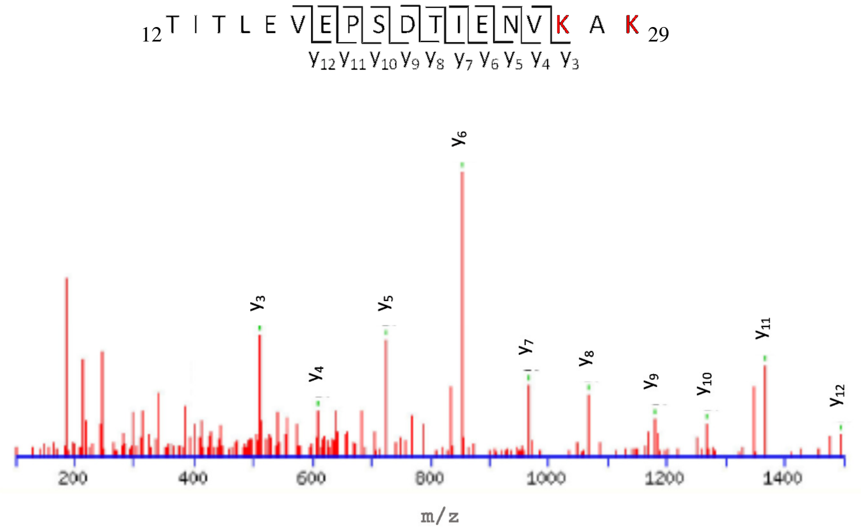

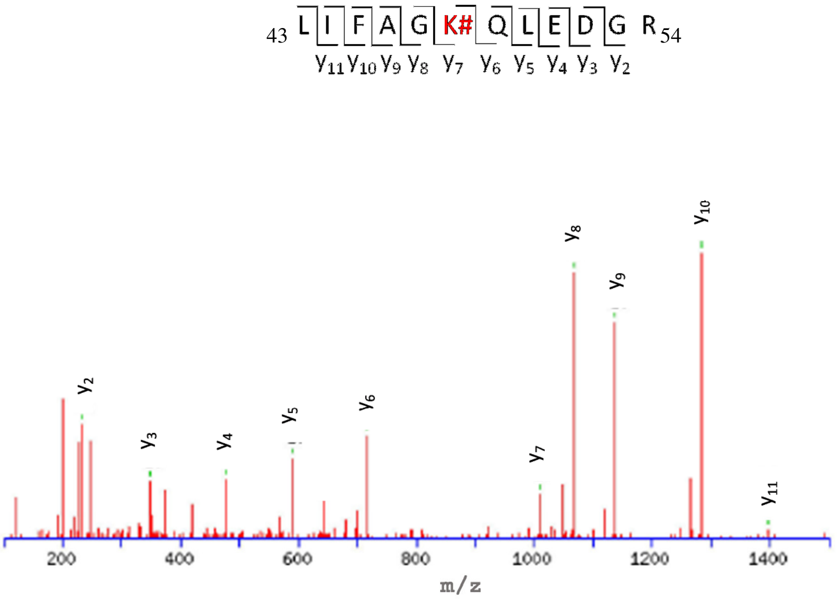

Figure S6. ESI-MS/MS spectra and fragmentation pathway of the HTB-modified peptide 3 (top) and 5 (bottom)

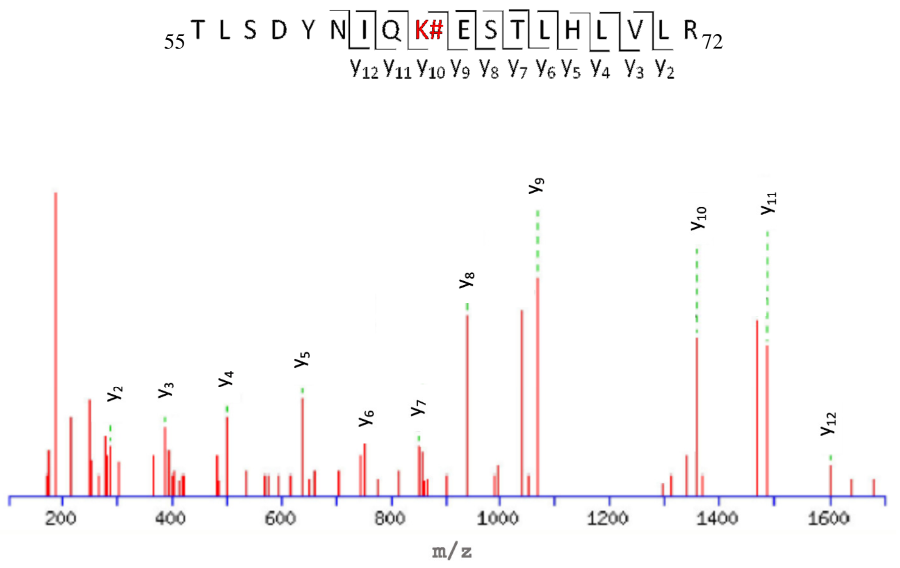

Figure S7. ESI-MS/MS spectra and fragmentation pathway of the HTB-modified peptide 6
